# Air Pollution: A Silent Key Driver of Dementia

**DOI:** 10.3390/biomedicines11051477

**Published:** 2023-05-18

**Authors:** Pawel Serafin, Malgorzata Zaremba, Dorota Sulejczak, Patrycja Kleczkowska

**Affiliations:** 1 Military Institute of Hygiene and Epidemiology, 01-163 Warsaw, Poland; pawelserafin1@wp.pl (P.S.); malgorzata.zaremba@wum.edu.pl (M.Z.); 2 Department of Experimental and Clinical Pharmacology, Centre for Preclinical Research (CBP), Medical University of Warsaw, 02-097 Warsaw, Poland; 3 Department of Experimental Pharmacology, Mossakowski Medical Research Institute, Polish Academy of Sciences, 5 Pawinskiego Str., 02-106 Warsaw, Poland; dots@op.pl; 4 Maria Sklodowska-Curie, Medical Academy in Warsaw, Solidarnosci 12 Str., 03-411 Warsaw, Poland

**Keywords:** air pollution, fine particulate matter, dementia, risk factors

## Abstract

In 2017, the Lancet Commission on Dementia Prevention, Intervention, and Care included air pollution in its list of potential risk factors for dementia; in 2018, the Lancet Commission on Pollution concluded that the evidence for a causal relationship between fine particulate matter (PM) and dementia is encouraging. However, few interventions exist to delay or prevent the onset of dementia. Air quality data are becoming increasingly available, and the science underlying the associated health effects is also evolving rapidly. Recent interest in this area has led to the publication of population-based cohort studies, but these studies have used different approaches to identify cases of dementia. The purpose of this article is to review recent evidence describing the association between exposure to air pollution and dementia with special emphasis on fine particulate matter of 2.5 microns or less. We also summarize here the proposed detailed mechanisms by which air pollutants reach the brain and activate the innate immune response. In addition, the article also provides a short overview of existing limitations in the treatment of dementia.

## 1. Introduction

Dementia, and the cognitive impairment that precedes it, is a common multifactorial disease with no singular cause, and its likelihood of occurrence increases with age. The globally aging population means that the absolute numbers of those living with dementia continue to increase with an estimated new case every three seconds [1].

There are currently more than 57 million people worldwide living with dementia, and it is one of the biggest global public health and social care challenges that people face now and will continue to face in the future [2]. By 2050, it is estimated that this number will rise to 131.5 million. Dementia also has a huge economic impact. It is estimated that the total global cost of dementia will reach USD 2 trillion by 2030 [1]. The fact that dementia is being diagnosed at an increasingly young age seems alarming. According to a new report by the Blue Cross Blue Shield Association (BCBSA), the number of commercially insured Americans between the ages of 30 and 64 diagnosed with early-onset dementia or Alzheimer’s disease (AD) jumped 200% from 2013 to 2017, from 4.2 to 12.6 per 10,000 [3]. 

There are several types of dementia, the most common of which is AD, a progressive neurodegenerative disorder that accounts for 60% to 80% of all dementia cases [4]. The key processes underlying AD include the accumulation of amyloid beta peptides into plaques, the formation of neurofibrillary tangles containing the tau protein, and neuronal degeneration. Other types of dementia include vascular dementia, dementia with Lewy bodies, dementia in Parkinson’s disease (PD), frontotemporal dementia (FTD), dementia in Huntington’s disease, and dementia in Creutzfeldt–Jakob disease [5]. Although each of these types is different, there are some standard features including the loss of basic brain features and capabilities. All types of dementia cause significantly impaired intellectual functioning. People with dementia lose their ability to solve problems, have an impaired ability to think abstractly and to plan and maintain emotional control, and may experience personality changes and behavioral problems such as agitation, delusions, and hallucinations. Over time, patients develop a severe phase, become totally dependent on their caregivers, and eventually die. Advances in understanding the role of the immune system, inflammation, and synapse degradation in dementia neuropathology are contributing to broad approaches to drug discovery and bringing hope for a clinical breakthrough in the causal treatment of neurodegenerative disease.

There are a huge number of potential risk factors for the development of dementia, including modifiable risk factors such as lifestyle, occupation, diabetes, physical inactivity, smoking, and obesity with a special place for genetic background (i.e., *APOE4* gene variant, *APP*, and *PSEN1/2* gene mutations) [6,7,8,9,10,11,12,13]. There are also plausible links between exposure to certain air pollutants and dementia. 

## 2. Current and Future Pharmacological Treatment Options for Dementia

**Figure 1:**
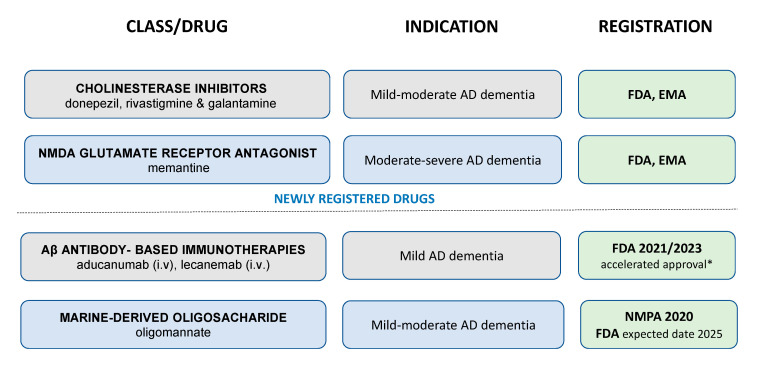
Current drugs approved for the treatment of cognitive impairment and AD-related dementia (registration and indication for oral and i.v. therapy). Abbreviations: AD—Alzheimer’s disease; i.v.—intravenous administration; Aβ—amyloid beta; NMDA—N-methyl-D-aspartate receptor; FDA—Food and Drug Administration (USA); EMA—European Medicines Agency (EMA); NMPA—National Medical Products Administration (China). * Continued approval will be based on further trials confirming a clinical benefit over currently available therapy.

Developing treatments for dementia is a challenge. The main drugs available for symptomatic treatment have only minimal and transient efficacy (Figure 1). None of them have been shown to slow the progression of the disease, and all carry the risk of life-threatening side effects and numerous interactions with other drugs [14,15,16,17]. The cholinesterase inhibitors donepezil, galantamine, and rivastigmine can cause side effects, including sometimes severe vomiting; cardiac symptoms, such as arrhythmia and conduction disturbances; bradycardia; collapse; and syncope. In addition, donepezil can cause compulsive sexual behavior [18,19]. Memantine, an NMDA glutamate receptor antagonist, can cause neuropsychiatric symptoms such as hallucinations and confusion, sometimes leading to violent behavior, seizures, psychotic disorders, and heart failure or bradyarrhythmia [20]. Nevertheless, until recently, AD treatment research has focused almost exclusively on amyloid plaques. A new category, aducanumab, which is an anti-amyloid-beta (Aβ) monoclonal antibody, received accelerated approval from the FDA in June 2021, a decision that marks the first approval of a new drug for AD since 2003. Aducanumab appears to have limited evidence for clinical benefit, pending the results of an ongoing Phase 4 trial. The newly registered drug is ineffective in treating individuals with advanced disease and seems to work better as a preventative treatment for people with mild cognitive impairment or mild stages of dementia (Figure 1). It is supposed to remove Aβ plaques that accumulate in the brain, although removing the plaques does not appear to dramatically slow the progression of the disease [21,22,23,24]. 

Lecanemab is the second Aβ monoclonal antibody approved in January 2023 by the FDA under the accelerated approval pathway for the treatment of mild cognitive impairment or mild stages of dementia. 

Thus, current Aβ antibody-based immunotherapies have an inherent risk of causing more harm than good due to the inflammatory side effects of amyloid-related imaging abnormalities (ARIA) and have numerous limitations, including the high cost of treatment. Aβ monoclonal antibodies may carry the risk of causing amyloid-related edema (ARIA-E) or hemosiderin deposition-related microhemorrhage (ARIA-H), which are known to occur with antibodies. ARIA most often manifests as temporary swelling and may be accompanied by small spots of bleeding in or on the brain, although serious and life-threatening events are rare [25]. Patients may experience symptoms such as headache, confusion, dizziness, vision changes, nausea, and seizures. Remarkably, in clinical trials, aducanumab was associated with encephalitis and bleeding in one-third of those who received the FDA-approved dose. Two patients in the treatment arm of the lecanemab clinical trials died from the side effect of strokes leading to brain hemorrhages [26]. In addition, warnings about lecanemab include a risk of infusion-related reactions, with flu-like symptoms, nausea, vomiting, and changes in blood pressure [27]. There remains an unmet need for other safe disease-modifying therapies for AD due to all the controversy surrounding the approval of aducanumab and the significant risks and limitations for both antibodies.

Given that the dysbiosis of the gut microbiota and the abnormal increase in intestinal flora metabolites can cause inflammation, the first marine-derived oligosaccharide to recondition the gut microbiota, oligomannate (GV-971), was approved in China in 2020 for mild-to-moderate AD (Figure 1). This oral drug reduces amyloid protein deposition and tau hyperphosphorylation via the peripheral and central modulation of dysbiosis-related inflammation, reducing the contribution of altered peripheral immunity to AD pathogenesis. GV-971 was also found to be safe and well tolerated without side effects typical of Aβ monoclonal antibodies [28,29]. Marketing applications in selected countries are planned. A multicenter global Phase III clinical trial (GREEN MEMORY) with sites in the USA, Europe, and Asia was conducted to support the global regulatory filing of oligomannate. Recently, GV-971 was given the green light from the FDA and has entered Phase III, with an expected registration date in other countries in 2025.

Gene therapy using neurotrophins called nerve growth factor (NGF) and brain-derived neurotrophic factor (BDNF), which support the survival of existing neurons and promote the growth and differentiation of new neurons and synapses, may be the future method for treating AD. Animal trials from 2001–2012 using AAV2 and NGF have provided promising features in this regard [30]. The main drawback of such neurotrophins is their limited crossing of the blood–brain barrier; thus, researchers have resorted to gene therapy, in which a modified adeno-associated virus (AAV2) more efficiently delivers and distributes the protein gene to key structures in dementia (entorhinal cortex and hippocampus). Based on the ongoing first-in-human Phase I clinical trial with AAV2-BDNF, this gene therapy represents an advancement over previous studies with NGF completed in 2010 [31]; thus, it appears to be a more potent growth factor than NGF for the neuronal circuits that are affected in AD the earliest [32]. It should be noted that BDNF injections must be precisely controlled to avoid side effects as freely circulating BDNF can cause seizures. The estimated completion date of the study is scheduled for the end of 2027.

Another ongoing Phase 2 study aims to evaluate the safety and toxicity of AAV gene transfer vector expressing the cDNA coding for human APOE2 (AAVrh.10hAPOE2) (LX1001) for the treatment of patients who are APOE4 homozygotes with mild cognitive impairment due to AD, and mild dementia [33]. Direct intrathecal administration of LX1001 to the CNS of these AD patients will lead to the conversion of APOE protein isoforms in the CSF of APOE4 homozygotes from APOE4 to APOE2-APOE4. The estimated completion date of the study is expected soon at the end of 2024.

It should be noted that there are currently no therapies that can effectively act on tau, so a gene-silencing drug is urgently needed to slow down—and perhaps even reverse—the course of Alzheimer’s disease and other diseases caused by tau accumulation. A significant step forward in this field is an ongoing world-first Phase I clinical trial published in Nature Medicine—with results from 46 patients—testing the safety of a drug called BIIB080 (/IONIS-MAPTRx), which is an antisense oligonucleotide (used to stop RNA producing the protein), to “silence” the gene encoding the tau protein, known as the tau microtubule-associated protein (MAPT) gene [34,35]. This prevents the gene from being translated into a protein in a dose–response and reversible manner. It also lowers the production of this protein and alters the course of the disease. The study looked at three doses of the drug, administered by intrathecal injection (injection into the nervous system via the spinal canal), compared to placebo. No serious adverse events occurred in patients who received the drug. The study found a greater than 50% reduction in CNS levels of total tau and tau phosphorus after 24 weeks in the two treatment groups that received the highest dose of the drug. There is a need for further studies evaluating the drug in older and larger groups of subjects and in more diverse populations. The estimated completion date of the study is scheduled for the end of 2026. 

In summary, drug development for AD faces challenges due to an incomplete understanding of the disease’s pathological mechanisms. Focusing solely on amyloids was simply a case of tunnel vision. Combination therapies targeting multiple pathological alterations would likely be more effective than single-target anti-amyloid therapies currently approved on the market.

## 3. The Non-Pharmacological Treatment Options for Dementia

Given that the medications still have strong limitations, non-pharmacological interventions (i.e., reassurance, increased activities, etc.) are commonly proposed, which are mainly aimed at improving patients’ cognitive function and learning to cope with psychological and behavioral symptoms of dementia such as depression, delusions, aggression, and others [36]. These activities can, in turn, significantly improve the quality of life and well-being of people living with dementia, including their families [37]. Based on suggestions made by Cammusuli et al. [37], these interventions can be divided into the following groups: (i) holistic techniques, (ii) primary psychotherapy, (iii) cognitive methods, and (iv) alternative methods. Holistic approaches have continuously been shown to benefit both dementia patients and their caregivers. Similarly, the aforementioned cognitive training, cognitive stimulation, and combinations of these interventions have been suggested to positively affect cognitive function in patients with dementia [38,39,40]. Aerobic and resistance exercises are great examples of activities that have beneficial effects on cognition [41]. However, some studies have shown it to be moderate and limited to a specific activity (e.g., dance, Tai Chi, cycling, and stretching), while others have found no significant differences between experimental and control groups [42,43,44].

Therefore, while both pharmacological and non-pharmacological treatments remain a challenge, it is better to prevent dementia than to treat the already existing disease. Before embarking on preventive strategies, however, it is important to consider the knowledge of factors that can either induce or increase the risk of dementia development.

## 4. Environmental Risk Factors for Dementia

The world is undergoing constant change, which is mainly observed in global climate change as a response to several elements, such as solar radiation and an increase in solar input to the earth, the burning of fossil fuels, electromagnetic fields, and others. As a result of human activity and the rapid development of industry and transportation, several pollutants are generated, including those that pollute the aquatic ecosystem, which, paradoxically, can threaten human health and life in the long term. Indeed, all these factors, referred to as environmental factors, have been found to play a major role in the risk of dementia development and progression [45].

For instance, the presence of various toxic heavy metals and other chemical elements can be detected during water quality analysis. These include aluminum, cobalt, iron, lead, iron, zinc, and copper. Each of these has been linked to the risk of dementia. Iron, for example, is known as an essential component for the efficient delivery of oxygen to every cell in the human body. However, excessive amounts of it can cause several undesirable pathways, including ferroptosis, defined as iron-dependent cell death driven by lipid peroxidation [46]. This process is now widely described as a risk factor for dementia associated with AD [46,47,48]. Moreover, the effect of iron-induced ferroptosis on the onset of cognitive impairment has been confirmed in various animal models [49,50,51]. In contrast, only one case–control study by Emard and colleagues on the role of cobalt in dementia has been suggested [52]. Importantly, this was true not only when cobalt is present in the environment but also when it is implanted in the body (such as in the case of a hip implant) [52,53]. Emard also revealed that several people living in areas with high concentrations of lead suffered from AD [52]. Most papers describe aluminum exposure as the major risk factor for dementia, however, studies show no association or conflicting results [54,55]. This discrepancy in results is related to the different forms of aluminum studied. Aluminum from drinking water accounts for only about 5% of total intake; thus, there is little work on this source of the element. In contrast, information on the role of aluminum present in cosmetics and personal care products is more extensive [56]. Nevertheless, the role of aluminum in the development of dementia is indisputable, especially given the work published in 2008 by Rondeau et al. [57]. Aluminum was shown to double the risk of dementia and triple the risk of AD in water drinkers, especially at a concentration above 0.1 mg/day. Another recently published study confirmed such a relationship as the aluminum residue was found in post-mortem brain samples of patients diagnosed with familial AD [58]. In addition, dialysis dementia, also known as dialysis encephalopathy syndrome (DES), has been characterized in patients with renal failure undergoing hemodialysis who used aluminum-containing tap water for dialysis fluids [59,60].

### Air Pollutants

Air pollution is contamination that sometimes results from natural processes in the world (e.g., biological processes, volcanic eruptions, and the chemical weathering of rocks or forest fires), but it can also be the result of direct human activity (e.g., chemicals, refining, metallurgy, or the chemical conversion of fuels). Currently, air pollution is the largest environmental risk factor for multiple complex mental and physical diseases with serious economic consequences in the form of increased medical costs and reduced productivity. According to World Health Organization (WHO) estimates, air pollution causes 4.3 million deaths annually [61]. Notably, outdoor exposure to PM with an aerodynamic diameter <2.5 µm (PM2.5) is the fifth leading risk factor for mortality worldwide [62]. Major factors that have been linked to the disease include ozone (O_3_); components of tobacco smoke (i.e., benzene, toluene, and formaldehyde); the PM present in motor vehicle exhaust fumes, which contains various compositions of black carbon, SO_4_^2−^, NH_4_^+^, and NO_3_^−^ and metallic components (K, Ca, Zn, Fe, Al, and Mg), as well as nitrogen dioxide (NO_2_) and other oxides of nitrogen, carbon monoxide (CO), sulfur dioxide (SO_2_), or polycyclic aromatic hydrocarbons (PAH), such as phenanthrene, benzo (a)pyrene, benzo (b)fluoranthene, benzo (a)anthracene, fluorene, fluoranthene, pyrene, chrysene, benzo (k)fluoranthene, dibenzo (a,h)anthracene, indene (1,2,3-cd)pyrene, and benzo (ghi)perylene [63,64,65,66,67].

**Figure 2:**
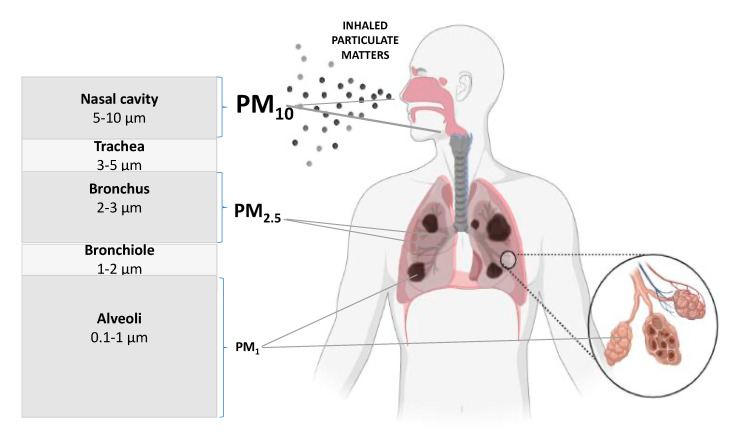
Size-dependent regional deposition of inhaled particulate matters. The deposition sites of various inhaled particles of differing size (PM10, PM2.5, and PM1), which are located in specific regions of the respiratory tract. Created with BioRender.com (accessed on 3 February 2023).

In 2017, the Lancet Commission on Dementia Prevention, Intervention, and Care included air pollution in its list of potential risk factors for dementia [68]; in 2018, the Lancet Commission on Pollution concluded that the evidence for a causal relationship between fine particulate matter and dementia is encouraging [69]. Currently, the most widespread human air pollution is PM, which is produced by so-called low emissions (i.e., any exhaust fumes entering the air at low altitudes). Particulate matter is a mixture of molecules for which harmful effects depend on the size of the particles [70]. In fact, three classes of PM can be distinguished: a particle with a diameter less than 10 µm (PM10), a particle with a diameter of less than 2.5 µm (PM2.5), and ultrafine particles (UFPs) with a diameter less than 0.1 µm (PM0.1) [70,71,72]. These are extremely fine particles: 2.5 μm, for example, is about one-thirtieth of the diameter of a human hair. It is known that the size and the fineness (dispersion) state of a particle have a key effect on its absorption mainly by the respiratory and gastrointestinal systems (Figure 2). While large particles settle in the nasopharynx and larynx, from where they can be easily removed, smaller particles, especially those below 5 µm, penetrate deeper into the body and accumulate in the lung bronchi; hence, they have easy access to the blood and have observed toxic effects [73] (Figure 2). However, the most health-damaging particles are even smaller. Given the above, PM0.1 is known to be responsible for inflammatory reactions in the airways, which can exacerbate the course of asthma or COPD (chronic pulmonary obstructive disease) [74]. In the case of PM, cytotoxicity is associated with increased levels of oxidative stress and the (neuro)inflammatory process [75,76,77]; however, each fraction may be characterized by its ability to induce its specific toxic effect [78,79]. 

The World Health Organization has estimated that more than four million deaths per year can be attributed to particles smaller than 2.5 μm in diameter [80]. The WHO offers guidelines (Air Quality Guidelines—AQG) for reducing the health effects of air pollution. Although the WHO AQGs state that annual average concentrations should not exceed 10 μg/m, almost ¾ of European countries still exceed the annual concentrations set by the WHO AQGs for PM2.5 pollution [81,82]. Boldo et al. found that in 23 European cities life expectancy at age 30 would increase from one month to more than two years if long-term exposure to PM2.5 levels were reduced to 15 μg/m [83].

## 5. Insights from Experimental and Clinical Studies

### 5.1. Animal Studies 

With regard to dementia, recently, Herr and colleagues reported that PM, particularly UFPs, increased tau phosphorylation in the hippocampus and enhanced microglial activity in a genetic 3xTgAD mouse model [84]. Tau phosphorylation has also been observed in other studies describing toxicity induced by higher PM2.5 [85,86,87]. Interestingly, neuronal loss, a common pathological phenomenon of neurodegeneration, has been observed in brain areas both in adult animals exposed to PM2.5 and in offspring after prenatal exposure during pregnancy [88]. 

### 5.2. Clinical Studies 

A prospective birth cohort study involving children found that black carbon was one of the PM components associated with cognitive decline, including memory impairment [89]. Consistently with these studies, Calderón-Garcidueñas [90] and other research groups [91,92] report that children who are chronically exposed to outdoor air pollution are characterized by early hallmarks of AD such as a significant increase in tau [92,93]. 

This seems significant considering the current Chinese diagnosis of the world’s youngest person with probable AD (age 19), who was found to have no genetic mutations [94]. Age currently remains the largest risk factor for AD, but this study aims to encourage more attention to exogenous, environmental risk factors for dementia of young onset.

Long-term PM exposure can also affect neurodevelopmental outcomes in children. However, it should be noted that most of the studies have been conducted in areas with a low level of PM. To the best of our knowledge, the NeuroSmog study will be the first to assess whether long-term outdoor exposure to PM affects brain structure, function, and connectivity in both healthy children (aged between 10 and 13 years) and those diagnosed with attention deficit hyperactivity disorder (ADHD) [95]. The study covers an area including 18 cities in Poland with the highest concentration of air pollution [95]. The planned use of an fMRI can provide detailed information on the functioning of neural systems with a particular focus on cognitive flexibility [96]. The authors hypothesized that children diagnosed with ADHD would be more vulnerable to increased exposure to air pollution. There is emerging evidence that greater exposure to air pollution is associated with an increased risk of dementia. Kioumourtzoglou et al. [97] found that exposure to PM2.5 increases the hospitalization of patients with AD. Similar findings were reported by Carey et al. [98] and in a newly published paper by Shi and colleagues [99], who provided information on the existing correlation between increased PM2.5 concentrations and higher levels of dementia. A large-scale cohort study that followed older adults for 10 years found that the risk of developing dementia associated with AD increases by nearly 140% as PM2.5 concentrations increase by 4.34 μg/m^3^ [100]. In contrast, Abolhasani et al. [101] found, based on 12 studies, that the risk of dementia increases by 3% for every 1 μg/m^3^ increase in PM2.5 concentrations. Similarly, three other cohort studies (NHS, WHIMS, and the Whitehall II longitudinal study) found a higher risk of cognitive decline associated with higher PM2.5 exposure [102,103,104]. In the NHS study, the rate of cognitive decline was significant in women with the highest level of PM2.5 exposure compared to the lowest level [104]. In one similar study, Cacciottolo et al. additionally noted a dose-dependent relationship between the apolipoprotein E4 (APOE4) allele and PM2.5, such that the smallest decline in cognitive function was in those with the lowest exposure and without an APOE4 allele [101]. Cleary et al. [105] also reported a dose–response relationship for the interaction between the presence of APOE4 and PM2.5 and and cognitive decline. Interestingly, Chen et al. [106] noted that the location of residence (near the road) appears to be another causal factor of cognitive decline. Based on their analysis, they analyzed the following parameters: a place of residence’s distance from the roadway, the level of both PM2.5 and NO_2_, and the risk of PD-related dementia. They found that living closer to a roadway was associated with an increased risk of PD-related dementia for all distance categories (1–100 m and 101–200 m) except for the distance of 201–300 m. In another population-based cohort study, the Betula Study, Oudin et al. [107] found an association between higher PM2.5 levels from traffic exhaust and incidence of dementia. Overall, these results showed a potential link to dementia and revealed PM as an agent with high toxicological potential in all age groups. It should be stated that there is no clear consensus as to what period of exposure is most informative for assessing the neurological effects of air pollution.

The results for nitric oxides, particularly NO and NO_2_, are inconclusive, and their involvement in dementia is not as proven as for PMs. For instance, one study found that NO is positively involved in learning and memory, while its inhibitors (i.e., L-arginine methyl analogs) have been linked to cognitive impairment in older adults [108,109]. On the other hand, some authors have suggested that elevated NO levels may result in abnormal protein modification and, thus, may be involved in the pathogenesis of some neurodegenerative diseases, including AD. Similarly, endothelial NO deficiency also leads to vascular endothelial dysfunction and cerebral hypoperfusion, which, in turn, may result in greater β-amyloid-induced damage [110,111,112,113]. Amyloids are self-aggregating proteins that can induce cellular dysfunction in patients at risk for neurodegenerative disorders. Nevertheless, a population-based retrospective cohort study of 1720 Taiwanese individuals exposed to various levels of NO_2_ between 1998 and 2010, found that increased NO_2_ exposure was associated with a higher risk of dementia in both sexes [114]. Moreover, a similar risk was widely observed in younger patients living in highly urbanized residential areas. In another cohort study, the authors reported that a higher risk of dementia was associated with a combination of air pollutants, such as NO_2_ and PM2.5 [115]. Abolhasani et al., in a systematic review and meta-analysis, suggested a nonsignificant association between dementia and nitrogen oxides, including NO_2_. This was mainly explained by the lack of a significant number of studies [101]. An interesting study was conducted by Wang and colleagues, who showed that improving air quality reduces the risk of dementia in older women [116,117].

Carbon monoxide is another important risk factor drawing attention. This poisonous gas, although non-irritating, is mainly found in car exhaust, but it is also in fumes produced by grills, heaters, or even fireplaces. Its main toxic effect is cerebral hypoxia and ischemia, as well as neurological defects, including dementia, parkinsonism, etc., which were observed in 10–30% of intoxicated patients [118,119,120,121]. In examining the potential link between dementia and CO, it has been suggested that the risk factor for dementia increases with age and the severity of intoxication [114,122]. Some studies have also reported a strong association between CO and dementia with additional noise exposure [123]. In fact, CO and noise co-intoxication were found to magnify the risk level. This was demonstrated by an increase in oxidative stress and ROS production. Another study showed a link between CO and dementia as CO was found to have the ability to induce APOE e4 (a genetic risk factor for dementia) in carriers of the gene with increased morbidity compared to non-carriers [124].

## 6. Mechanisms Leading to Dementia after Exposure to Air Pollution

**Figure 3:**
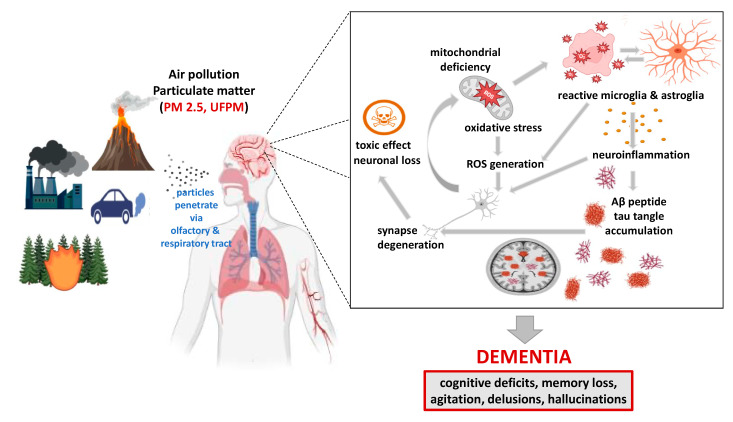
Environmental exposures to air pollutants in the etiology and pathogenesis of dementia. Sources of air pollution (fine and ultrafine particles—UFPM) include particles smaller than 2.5 μm in diameter, for which annual average concentrations should not exceed 10 μg/m. Air pollution is a common environmental factor that affects the brain through multiple pathways (i.e., through the olfactory tract and blood vessels via the respiratory system). Particles affect the brain by accelerating (priming) microglia and astroglia and, thus, initiating neuroinflammation, production of reactive oxygen species (ROS), deposition of amyloid beta (Aβ) peptides, and tau phosphorylation. Aβ causes synaptic impairment, neuronal death, and progressive neurodegeneration, ultimately leading to Ad-related dementia and cognitive impairment. Created with BioRender.com (accessed on 12 May 2023).

The fine PM and UFPs of air pollution described above are particularly hazardous to health. Due to their small size, they can reach and deposit in both the respiratory tract, the lungs (Figure 2) and the gastrointestinal tract. From there, they can enter the blood vessels [125]. Munzel and colleagues [126] showed that PM2.5 can damage vascular endothelial cells and lead to vascular dysfunction. Interestingly, UFPs can enter the brain directly through the olfactory nerve; thus, the human nose may be a port for the entry of air pollutants into the brain [127,128,129] (Figure 3). The penetration of even a few particles into the brain parenchyma triggers several defensive phenomena, including the induction and activation of microglia and then astroglia cells, and leads to the development of inflammation (Figure 3). Microglia are reprogrammed to a heightened proinflammatory state (priming) to produce more cytokine and become deleterious. The toxic activation of microglia might lead to aberrant synapse elimination in older age, which is part of the pathway to dementia [130]. The over- and under-expression of pro- and anti-inflammatory cytokines alter the homeostasis of the central nervous system (CNS) and may contribute to progressive neuronal dysfunction [131]. Two recent studies in mice and rats showed that astrocyte function and mitochondrial activity in the cortex were severely impaired by PM, with greater effects observed with the exposure to smaller particle sizes [132,133].

Recently, a clear link has been shown between the presence of UFPs and damage to the neurovascular unit in various brain structures [134,135]. The neurovascular unit is a complex morphological and functional structure that includes neuronal (neurons and interneurons), vascular (endothelial cells and pericytes) and glial cells (astrocytes), the basal lamina, and components of the extracellular matrix. It is the smallest functional unit of the brain responsible for the integrity of the BBB and the regulation of cerebral blood flow, thus ensuring proper brain function [136]. Damage within the neurovascular unit is known to lead to inflammation and neurodegeneration. Additionally, the vascular effects and red blood cell damage caused by UFPs can lead to neuronal degeneration and the development of dementia by causing brain inflammation or thrombosis, among other conditions [137,138]. Remarkably, a robust link between PM2.5 and vascular dysfunction has been noted [128]. Particles of this size can cause damage to endothelial cells in the brain; thus, such pathways may promote various types of dementia [137]. Direct cellular damage caused by air pollutants is high as they induce oxidative stress in the cell and damage mitochondria (Figure 3). The latter increases the intensity and overload of autophagy and mitophagy processes. This can lead to fragmentation and the inability to remove damaged mitochondria, as well as the degeneration of synapses and whole neurons [139] (Figure 3). However, the main mechanism of cell damage by fine PM and UFPs is based on the induction of free radicals, which destroy cellular structures and lead to the development of neuroinflammation and protein aggregation [140] (Figure 3). Air pollutants also induce abnormal neovascularization and the excessive autophagy of nerve cells [140]. Different types of proteins can bind to the surface of UFP, which can help air pollutants enter the body’s cells and interact with cell organelles [141]. Inside the cell, the particle matter can damage cellular proteins, interfere with protein folding and assembly processes leading to the formation of abnormal protein deposits [142], and damage the endoplasmic reticulum necessary for protein production [143]. An additional problem is magnetic particles, which become highly concentrated in the cell’s endosomes. They exhibit strong magnetic interactions and are sensitive to external magnetic fields, which can lead to the strong heating of the particles [144]. Many data show a strong link between exposure to air pollution and the development of cardiovascular diseases and events such as myocardial infarctions, transient ischemic attacks, and strokes [145,146,147,148,149,150,151]. All these pathologies, if the patient survives, can lead to the induction or worsening of dementia.

Air pollution can also affect the gut microbiome [152]. A normal gut microbial flora has a beneficial effect on digestive processes and immune function and is important for the proper functioning of the entire body, including the brain [137,153,154,155]. Fine and ultrafine particles damage not only the gut microbiome but also the neurons that make up the enteric nervous system, and through the vague nerves causing toxic effects on the CNS [156,157].

Air pollution particles may also cause local damage to body organs, leading to inflammation and oxidative stress [156,157]. Such local and, especially, systemic inflammation and oxidative stress can, in turn, lead to the progression of neurodegenerative diseases and dementia [125] (Figure 3).

Selected air pollutants and their potential effects on dementia onset are summarized in Table 1.

## 7. Limitations

The review presented here does not provide the full context of air pollution and dementia due to the numerous limitations of the included studies listed below. First, for the current study, this is not a systematic review, due to methodological diversity in the assessment of air pollution exposure in the included articles, which made the data too inconsistent to be combined into a single metanalysis. The evidence from the toxicological studies in animal and cellular models is limited and inconclusive. While there are a growing number of neuroimaging studies on the effects of air pollution exposure on dementia in the brain, they remain elusive. Moreover, data on measures linking air pollution exposure to other forms of dementia, i.e., vascular and frontotemporal dementia, are too limited. In fact, there is only one study devoted to dementia associated with Parkinson’s disease [106]. Therefore, dementia related to Alzheimer’s disease was the focus of this review. In addition, the measure of cognitive decline used in the study’s methodology does not in itself necessarily indicate an ongoing degenerative process and dementia. In addition, some studies considered multiple pollutants at the same time; thus, this does not allow for a direct causal relationship between dementia risk and air pollution. Finally, the data are mainly from population-based cohort studies of low quality and reliability, and the risk of moderate bias means that the results obtained in this way should be interpreted with caution. There is a need for more high-quality data with a larger number of study groups.

## 8. Conclusions and Future Directions

The causality of dementia is multifactorial, but air pollution may be a modifiable key factor in increasing individual risk by accelerating age-related changes observed in the brain. The impact on the CNS is chronic, beginning in childhood, and the pathology can take time (years) to accumulate. Intensive global efforts to improve air quality, in the form of long-term policies to reduce air pollution, have been successful in many regions, but half of the world’s population is still exposed daily to particulate pollution above the recommended standards. Given the global scale of dementia, and an aging population, only the increased control of fine particulate emissions and the implementation of innovative public health initiatives can minimize this risk and prevent dementia from reaching epidemic status in the future.

There is an urgent need to characterize the link between the chronic exposure to air pollutants and the risk of developing dementia and its implications for public health worldwide. In addition, understanding the precise mechanisms by which PM affects the body’s organs will allow for the better treatment of patients who develop symptoms related to its exposure. More neuroimaging and molecular data are needed to determine the cellular event that triggers the pathological brain response.

## Figures and Tables

**Table 1:** Major air pollutants and the type of their effects on the development and progress of dementia.

Air Pollutant	Effect	References
PM10	The relationship between PM10 and dementia is less clear, and the number of primary studies is more limited.	
PM2.5	↑ Tau hyperphosphorylation in the hippocampus and amyloid-β (Aβ) plaques;∙ Oxidative stress (shown as cytotoxicity and an abnormal proliferation in astrocytes) and microglia-dominated neuroinflammation (e.g., ↑ the serum and CSF neurofilament light (NEFL) polypeptide); Modification of endothelial cell miRNA; ↑ Blood pressure leading to vascular dementia; Modification of the gut–brain axis; Increased *DNA* methylation and, thus, MtDNA damage; ↓ White matter volume in the frontal lobe, temporal lobe, and corpus callosum; White matter atrophy.	[75,76,77,137,158,159,160,161,162,163,164,165,166,167]
UFPs (e.g., PM0.1)	↑ Tau hyperphosphorylation in the hippocampus and amyloid-β (Aβ) plaques; Microglia modification (ameboid vs. ramified); Contribute to Alzheimer’s disease development by translocation to the cortex regions, where Alzheimer’s disease is initiated (cross the BBB); Oxidative stress (which leads to increased permeability of the BBB); Neuronal inflammation (↑ excitatory neurotransmitters and proinflammatory cytokines) and degeneration as well as oligodendrocyte dysfunction; Ventriculomegaly.	[84,118,125,131,158,159,168,169]
Ozone	The relationship between ozone and dementia is unclear. Several papers have demonstrated that O_3_ exposure per se may not cause AD, but it can synergize with genetic risk factors to accelerate the pathophysiology of AD. Nonetheless, some effects were noted, and these include the following: Oxidative stress (e.g., lipid peroxidation in the hippocampus and cortex in vivo); Vascular dementia; Cellular destruction, swelling, inflammation, and mitochondrial changes; ↑ Protein serum amyloid beta.	[170,171,172,173]
Nitrogen dioxide	Beta-amyloid aggregation and plaque formation; Neuronal damage.	[174,175]
Polycyclic aromatic hydrocarbons (PAH)	Since PAHs are associated with particulate matter of an aerodynamic diameter ≤2.5 μm (PM2.5), it is not obvious whether PAHs solely can be associated with the development and/or progression of dementia. However, a body of evidence confirmed such a relationship, and this includes the following: Oxidative stress and inflammation;	[175,176]

The effects summarized for PMs, including UFPMs, were not categorized based on PM’s composition (such as sulfate, nitrate, ammonium, organic carbon, elemental carbon, and heavy metals), surface properties, and concentration. ↑ = increase; ↓ decrease.

## Data Availability

Not applicable.
